# Psychometric properties of two ADHD rating scales used in children with ADHD and intellectual disability

**DOI:** 10.1111/jir.13185

**Published:** 2024-09-07

**Authors:** M. Palmer, Z. Fang, V. Carter Leno, E. Simonoff

**Affiliations:** ^1^ Department of Child & Adolescent Psychiatry Institute of Psychiatry, Psychology & Neuroscience (IoPPN), King's College London London UK; ^2^ Centre for Brain and Cognitive Development Birkbeck, University of London London UK; ^3^ Service for Complex Autism and Associated Neurodevelopmental Disorders South London and Maudsley NHS Foundation Trust London UK

**Keywords:** ADHD, autism, instrument performance, intellectual disability

## Abstract

**Background:**

Attention deficit hyperactivity disorder (ADHD) is often present in people with intellectual disability (ID) and autism. However, few ADHD measures have been developed specifically for individuals with these conditions. There is little literature exploring how well ADHD measures are performing at picking up specific symptoms at the item level.

**Methods:**

Analyses were conducted on data from 122 children aged 7–15 years old with diagnoses of both ADHD and ID enrolled in the Hyperactivity and Special Educational Needs trial. Parents and teachers completed ratings of ADHD symptoms on the Aberrant Behavior Checklist (ABC) hyperactivity subscale and the revised Conners' Rating Scales hyperactivity scale and ADHD index. Cronbach's alpha was used to examine the reliability of these measures. Item response theory explores the performance of individual items. Multiple indicators, multiple causes models were used to test for measurement invariance by ID severity, co‐occurring autism traits and child age.

**Results:**

The reliability of parent and teacher reports of ADHD symptoms on the Conners' and ABC was acceptable across the range of ID. Item performance was generally good, and information was provided across the continuum of ADHD traits. Few items on either measure were non‐invariant (i.e., item endorsement generally did not differ based on other child characteristics). When non‐invariance was found, the effect was small.

**Conclusions:**

Both the parent‐reported and teacher‐reported versions of the Conners' hyperactivity scale and ADHD index and the ABC hyperactivity subscale appear to function well in the current sample of children with co‐occurring ADHD and ID.

## Introduction

Attention deficit hyperactivity disorder (ADHD) is characterised by problems with inattention and hyperactivity/impulsivity that are greater than expected for an individual's age and developmental level (American Psychiatric Association 2013). ADHD in childhood is typically assessed through reports from multiple informants spanning more than one context (usually parents and teachers) about a child's symptoms, either using validated questionnaires or using diagnostic interviews. It can often be a challenge to put together information from different sources to understand the full picture of a child's needs, particularly when reports differ between parents and teachers. Furthermore, ADHD frequently co‐occurs with other neurodevelopmental conditions such as intellectual disability (ID) and autism spectrum disorder (hereafter referred to as autism, a preferred term amongst the autistic community; Kenny *et al*. 2016). For example, Kadesjö and Gillberg (2001) report rates of 13% and 7%, respectively. ADHD is also more prevalent in these populations than in the neurotypical population (Buckley *et al*. 2020; estimates of 30–40% are reported; Rong *et al*. 2021). This co‐occurrence of conditions adds to the complexity of diagnosing ADHD and may lead to diagnostic overshadowing (Mason & Scior 2004) ‐ behaviour associated with ID, such as the length of time an individual can sit still and concentrate on a task, may be misattributed to the ID rather than ADHD. Similarly, autism symptoms (e.g., difficulties with shared attention) may also look like ADHD symptoms and be attributed to autism rather than co‐occurring ADHD.

Despite the prevalence of co‐occurring conditions, most questionnaires used to measure ADHD symptoms have been developed for the general population (i.e., without co‐occurring ID or autism). How the presence of these co‐occurring conditions impacts the performance of existing tools to measure ADHD is not well understood. Understanding whether these frequently co‐occurring characteristics impact the reliability and validity of ADHD questionnaires has important clinical implications. Such measures are often used to screen for ADHD symptomatology, guide diagnosis and monitor the effect of ADHD treatments (e.g., behavioural interventions or psychostimulants). Previous research exploring the accuracy and reliability of ADHD screening instruments in ID samples has reported mixed results. Our systematic review (in preparation) of the accuracy of ADHD screening instruments used with children with ID/autism found that although overall screening instruments had good diagnostic accuracy, no single tool met minimal criteria for adequate sensitivity and specificity simultaneously, albeit with a sample of four studies. Other research reported adequate internal reliability for a range of parent and teacher ADHD screening instruments amongst forty‐eight 5 to 12‐year‐olds with ID (Miller *et al*. 2004). Teacher reports were generally more reliable than those of parents. Deb *et al*. (2008) examined the diagnostic utility of the Conners' with children with ID and found that, in contrast, the parent version, but not the teacher version, may distinguish between intellectually disabled children with and without ADHD. However, many items were not applicable to children with severe or profound ID who have limited verbal communication (Deb *et al*. 2008). Therefore, further research is needed to explore how well specific items are at detecting ADHD symptoms in children with ID. In addition, Deb *et al*. (2008) also reported that parent and teacher reports were not correlated in their sample. The low correlation between parents and teachers may be due to situational specificity; that is, reports of behaviour could be influenced by the nature of the settings in which different informants interact (De Los Reyes *et al*. 2013). However, rater biases cannot be ruled out.

Other research has also explored whether ADHD rating scales are measurement invariant (i.e., whether endorsement of an item varies according to another variable). Arias *et al*. (2019) explored the item functioning of inattention and hyperactivity/impulsivity symptoms measured using the Child and Adolescent Behaviour Inventory (Cianchetti *et al*. 2013) amongst a sample of 7 to 15‐year‐olds with and without mild to moderate ID. Most items were measurement invariant; however, four items were less psychometrically accurate with ID children, such as ‘*losing things*’ (endorsed more often) and ‘*talking too much*’ (endorsed less often). The authors suggested that expressive language and environmental supports may impact the opportunity for children with ID to demonstrate these behaviours and therefore influence responses to these items. Furthermore, some of these items (e.g., ‘*loses things*’) had a substantial effect on diagnosis, suggesting that binary symptom counts should not be used. A key limitation of Arias *et al*. (2019) was that children with severe and profound ID were excluded under the assumption that they were more likely to be aetiologically and qualitatively different from those with mild to moderate ID (Reichenberg *et al*. 2016). This means that we cannot generalise these findings to all children across the ID spectrum.

The present study aimed to examine the psychometric properties of two widely used instruments for assessing ADHD; one developed for use with individuals in the general population and the other for those with developmental disabilities. We tested the psychometric properties of the revised Conners' Rating Scales hyperactivity scale and ADHD index (Conners *et al*. 1998a) and the Aberrant Behavior Checklist (ABC; Aman 2013) hyperactivity subscale in a sample of children with ID. Specifically, we examined the internal reliability of the two scales and then used item response theory (IRT) to evaluate the severity, discrimination ability and information provided from the different questionnaire items. This enabled us to examine the precision of the measurement of ADHD symptoms for each item, providing information about how ADHD manifests in children with co‐occurring ID. We then tested for measurement invariance, looking at whether a child's age, intelligence quotient (IQ) or level of autism symptoms influenced the likelihood of endorsing specific items on the scales. This provided us with information as to whether certain items are influenced by these characteristics, which may in turn guide decision‐making about screening and diagnosis. As parent and teacher ratings are often both collected during clinical evaluations, we examined the psychometric properties of both parent and teacher reports.

## Methods

### Sample and procedure

The sample was 122 children aged between 7 and 15 years who participated in the Hyperactivity and Special Educational Needs trial (a double‐blind randomised controlled trial of methylphenidate for children with ADHD and ID; see Simonoff *et al*. 2013 for further details). Only data from screening and baseline assessments are used in the current study (see Table 1 for sample characteristics).

**Table 1:** Sample characteristics

Characteristic	*N* = 122
Child	
Mean age in years (*SD*), range	11.16 years, (2.37), 7–15 years
Sex: *N* (%) male	85 (70%) male
School type: mainstream*, specialist, other: *n* (%)	33 (27%) mainstream, 84 (69%) specialist, 5 (4%) other
Mean IQ (*SD*), range	53.45 (10.05), 31–71
IQ groups: *N* (%)	39 (32%) moderate or greater (IQ < 50), 83 (68%) mild (IQ ≥ 50–70)
Mean SCQ (*SD*)^†^, range	16.80 (8.02), 0–33
Autism groups: *N* (%)	47 (44%) autism unlikely (SCQ < 15), 26 (24%) possible autism spectrum disorder (SCQ ≥ 15 < 22), 35 (32%) very likely to have autism
Parent	
Informant: mother, father, other^‡^: *n* (%)	104 (86%) mothers, 10 (8%) fathers, 7 (6%) other
Parental education: GCSEs or lower^§^: *n* (%)	67 (56%)
Teacher	
Informant: class teacher, learning support assistant, other^¶^: (*n*), %	89 (78%) teachers, 10 (9%) learning support assistant, 15 (13%) other
Time known child: less than one term, more than one term^‡^: (*n*), %	9 (7%) less than one term, 112 (93%) more than one term

* Mainstream education provision refers to mainstream education or a special unit in mainstream educational setting. Specialist refers to special educational provision (for learning disabilities, emotional and behavioural difficulties or physical disabilities).

^†^ *n* = 108.

^‡^ *n* = 121.

^§^ *n* = 119.

^¶^ *n* = 114.

IQ, intelligence quotient; SCQ, Social Communication Questionnaire; GCSEs, General Certificate of Secondary Education.

Participating children were recruited from clinical referrals (60% of the sample) and community screening (40% of the sample, through screening of special educational needs registers and approaching individual special schools) across Southeast England. All participating children had 1) a gold standard research diagnosis of International Classification of Disease‐10 hyperkinetic disorder based on the parent‐reported Child and Adolescent Psychiatric Assessment (Angold *et al*. 1995, 2) direct behavioural observations conducted during a research visit, which included IQ testing, 3) and parent/teacher questionnaires (Conners' as described below) completed as part of the trial. All information contributing to a diagnosis was written up in a vignette and reviewed by a senior clinician experienced in ADHD diagnosis in those with ID (see the supporting information for a full description of how the ADHD diagnoses were operationalised). As the study was conducted before the Diagnostic and Statistical Manual of Mental Disorder (DSM)‐V, which was the first time co‐occurring autism and ADHD were allowed, autism diagnoses were not made. However, we appreciated the need to explore autistic symptoms in this population and included the measure described below to assess for possible co‐occurring autism. Additional inclusion criteria for the trial were having a full‐scale IQ of 30–69, having a stable living situation and regular school attendance. Exclusion criteria were a severe mobility limitation, current use of stimulants or sensitivity reaction to stimulants, use of neuroleptic medication in the previous 6 months, diagnosed dementing disorder, epilepsy with daily seizures, presence of psychotic, bipolar, severe obsessive‐compulsive disorder or severe Tourette syndrome, risk of suicidal or homicidal behaviour, ongoing child protection concerns or having a household resident with current substance abuse disorder.

Written informed consent was obtained from the parents/guardians of the children and formal assent was obtained from older children and those with mild or moderate ID. Approval for the trial was granted from the Southeast Multi‐Centre Research Ethics Committee (MREC +04/01/013) and the Medicines and Healthcare Products Regulatory Authority (MHRA 8000/13629). The trial was registered on the International Standard Randomised Controlled Trial Number Register (ISRCTN68384912).

### Measures

Parents completed the revised Conners' Parent Rating Scale, from which the hyperactivity scale and ADHD index are derived (Conners *et al*. 1998a). The hyperactivity scale consists of six items measuring hyperactivity and impulsivity on a 4‐point scale. The ADHD index is made up of 12 items tapping into symptoms of inattention and hyperactivity/impulsivity on a 4‐point scale. Higher scores on both scales indicate greater symptomatology. In addition, teachers completed the Conners' Teacher Rating Scale (CTRS‐R version), from which a 7‐item hyperactivity scale and 12‐item ADHD index were calculated using the same 4‐point scale (Conners *et al*. 1998b). Both parent and teacher versions of the Conners' are widely used in research and clinical practice to measure ADHD symptomatology. They have good psychometric properties and discriminative validity in samples of children without ID (Conners *et al*. 1998a, 1998b).

Parents and teachers also completed the ABC (Aman 2013) hyperactivity subscale. The ABC was developed specifically for use with individuals (adolescents/adults) with developmental disabilities, and the hyperactivity/non‐compliance subscale (referred to throughout as ‘hyperactivity’) has 16 items that tap into symptoms of hyperactivity and non‐compliance. Items are rated on a 4‐point scale with higher scores indicating more hyperactivity. The same questions are used for parents and teachers. The ABC has demonstrated adequate psychometric information in children with developmental disabilities (Rojahn & Helsel 1991; Norris *et al*. 2019). It is often used in trials evaluating interventions for ADHD symptoms.

Intellectual ability was measured using the Mullen Scale of Early Learning (Mullen 1995), the Wechsler Preschool and Primary School Intelligence‐III Scales (Wechsler 2002) or the Wechsler Intelligence Scales for Children‐IV‐UK (Wechsler 2004), depending on the child's age and estimated ability. Three children completed the Mullen, 46 completed the Wechsler Preschool and Primary School Intelligence and 73 completed the Wechsler Intelligence Scales for Children. When standard IQ scores could not be obtained because a chronological age‐norm assessment would produce floor effects (i.e., if it became apparent during testing that the test was not accessible largely due to significant ID), tests were used out of age range to obtain a more accurate measure of a child's ability. A ratio IQ score was then calculated using the following formula: (IQ test age‐equivalent/chronological age) × 100. For comparisons across the range of ID, the sample was split into two subgroups: those with a mild ID (full‐scale IQ 50–70, *n* = 83, referred to hereafter as the mild ID group) and those with moderate/severe ID (full‐scale IQ < 50, *n* = 39, referred to hereafter as the severe ID group), as per commonly used clinical and research guidelines (Simonoff 2015).

Parent‐reported autism traits were measured using the total score from the Social Communication Questionnaire – Lifetime version (SCQ, Rutter *et al*. 2003). This version consisted of 39 items that assessed the presence/absence of a range of autism symptoms throughout the child's lifetime. The SCQ is a validated screen for autism symptoms used widely in both research and clinical settings and is associated with an autism diagnosis (Charman *et al*. 2007). Scores greater than or equal to 15 indicate a possible autism spectrum disorder, and scores greater than or equal to 22 indicate possible autism.

### Analysis

#### Analysis approach

This secondary data analysis was conducted to assess internal reliability, item performance and measurement invariance of the various parent‐reported and teacher‐reported hyperactivity/ADHD measures in Stata Release 17 (StataCorp 2021).

#### Reliability

Cronbach's alpha coefficient (Cronbach 1951), the item‐total correlations and the alpha if item omitted were used to evaluate the internal consistency of the scales. A Cronbach's alpha of ≥0.70 is considered acceptable (Taber 2018). We assessed internal reliability in the full sample as well as the ID subgroups, as we wanted to explore if internal reliability differed across the spectrum of ID.

#### Item response theory

Item response theory (IRT) models are an alternative statistical approach to classical test theory (Jabrayilov *et al*. 2016). They aim to indirectly measure a latent trait (*θ*) by examining the estimations of the probability of endorsing an item, providing person parameter invariance and information about measurement precision that the scale captures. This means that information on whether an informant will endorse any item is extracted from these analyses, adding to the understanding of which items are most useful or relevant for that informant in assessing ADHD. When inferring trait levels, it takes the pattern of item scores into account. In classic test theory, it is assumed that measurement precision is equal for all, regardless of their levels of symptoms. In IRT, the severity, discrimination ability and information of the items are evaluated (see Table 2 for definitions of these terms). In the current study, the two‐parameter item response theory (2PL‐IRT) model (Baker 2001) was used and applied to the whole sample. The 2PL‐IRT model provides estimates of how the specific characteristics of the items function in relation to the latent trait. It models the probability of symptom endorsement based on the severity (difficulty) and discrimination parameters. As the 2PL‐IRT model requires binary data, the response options were split into two categories. The analysis was first run for each cut point on parent data (i.e., 0 vs. 1+, 0–1 vs. 2–3 and 0–2 vs. 3). The results were visually examined to determine which model best fitted the data. For the current study, the cut point of 0–2 vs. 3 was used for both parents and teachers for consistency across informants. For ease of presentation, the items were split into two groups in cases where there were too many items to interpret from one graph. Subgroup IRT analyses were not possible, as it would have reduced sample sizes by too much to be confident in the findings.

**Table 2:** Glossary of key terms related to item response theory and measurement invariance

Terminology	Definition	Example
Item discrimination ability, discrimination parameter	This refers to how well the symptom can discriminate between different levels of hyperactivity/ADHD across the trait. The steepness of the S‐shaped curve in the item characteristic curve (ICC) graphs indicates the item discrimination ability.	How well the item discriminates between those with high and low scores on *inattentive, easily distracted*.
Item severity, difficulty (severity) parameter	Severity refers to the amount of hyperactivity/ADHD required for an item to be endorsed at different levels of the trait. The parameter signifies the level of trait required for a 50% chance of a symptom being endorsed. The position of the S‐shaped curve in the ICC graphs across the trait (x axis) indicates the item severity.	How *inattentive, easily distracted* an individual is.
Item information	The item information refers to how precise or reliable an item is as an indicator of hyperactivity/ADHD across the trait. The position and how peaked the bell‐shaped curves in the item information function (IIF) graphs indicate the amount of information provided by the item on the trait continuum.	Endorsement of *inattentive, easily distracted* may be informative for low scorers, but not for those with high scorers.
Multiple indicators, multiple causes (MIMIC) model	The MIMIC model examines the influence of a continuously measured covariate on item endorsement of symptoms of a latent trait. By default, it will test invariance at the scalar level.	For the same levels of hyperactivity/ADHD, does endorsement or *inattentive, easily distracted* vary by intellectual ability (IQ scores)?
	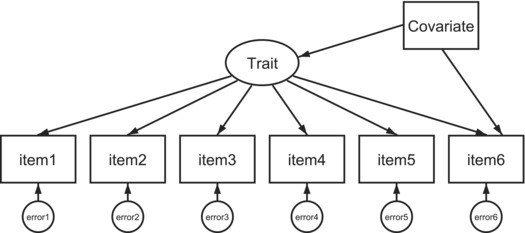 Diagram depicting the structure of the MIMIC model used to test for measurement invariance. Here, the latent trait is measured through each item on the measure (indictors), and there is an error attached to each individual item. The covariate is the proposed cause that is thought to influence the unobserved latent trait. In such a model, the impact of the covariate on the measurement of the trait is being tested. For ease of presentation, only a path is drawn from the covariate to item 6, but the impact on all items is tested.	

ADHD, attention deficit hyperactivity disorder; IQ, intelligence quotient.

#### Measurement invariance

Measurement invariance tests whether the items (indicators) assess the same latent trait in the same way according to the specified covariate (Bauer 2017). There are four successive levels of measurement invariance. They are configural (the latent trait and same number of items apply), metric (the items relate to the latent trait in the same way), scalar (the same item response is expected for the same amount of the latent trait) and residual (the latent trait explains the items equivalently). In the current study, we used multiple indicators, multiple causes (MIMIC) models to explore whether, given the same level of ADHD symptoms, the specific items proposed to measure the latent trait would vary according to a given covariate (see Table 2 for a diagram). The MIMIC model was used because it gave us greater statistical power to detect effects by treating each covariate as a continuous factor. We explored the severity of ID, the levels of co‐occurring autism symptoms and child age as possible covariates of item endorsement for each measure using MIMIC models. We conducted backwards stepwise selection, constraining each item by the largest *p*‐value.

## Results

### Reliability

#### Parent measures

Reliability was acceptable for the parent Conners' hyperactivity scale for the whole sample and when split by ID subgroups (see Table 3 for means and *SD*s and alphas). None of the items were problematic according to the item‐total correlations or alpha if item deleted (Table 3). For the parent Conners' ADHD index, Cronbach's alpha was acceptable for the whole sample and for ID subgroups. For those with severe ID, no items were problematic according to the item‐total correlations or alpha if item deleted. For the sample overall and amongst those with a mild ID, the item *attends if interested* was weakly correlated with the total ADHD index score (0.38 and 0.28, respectively), but little improvement in alpha was observed if this item was removed (from 0.84 and 0.83 to 0.85 and 0.85, respectively).

**Table 3:** Means, standard deviations and Cronbach's alpha for the parent‐reported and teacher‐reported Conners' hyperactivity scale and ADHD index and the ABC hyperactivity subscale

Measure	*M* (*SD*)			Cronbach's alpha		
	IQ < 50	IQ 50–70	Total	IQ < 50	IQ 50–70	Total
Parent Conners' hyperactivity scale*	11.82 (4.13)	12.14 (3.72)	12.04 (3.84)	0.80	0.76	0.77
Parent Conners' ADHD index*	27.61 (6.64)	27.59 (5.54)	27.60 (5.89)	0.87	0.83	0.84
Parent ABC hyperactivity subscale^†^	32.82 (9.09)	30.83 (9.28)	31.47 (9.23)	0.89	0.90	0.90
Teacher Conners' hyperactivity scale^‡^	10.67 (6.15)	9.48 (5.77)	9.86 (5.89)	0.92	0.90	0.91
Teacher Conners' ADHD index^‡^	22.32 (9.85)	19.84 (9.27)	20.62 (9.49)	0.94	0.93	0.94
Teacher ABC hyperactivity subscale^†^	23.87 (14.06)	19.53 (12.96)	20.92 (13.44)	0.96	0.96	0.96

* *N* = 118–121, IQ < 50: *n* = 38–39, IQ 50–70: *n* = 80–82.

^†^ *N* = 120–121, IQ < 50: *n* = 38–39, IQ 50–70: *n* = 81–82.

^‡^ *N* = 118–120, IQ < 50: *n* = 37–38, IQ 50–70: *n* = 81–82.

ADHD, attention deficit hyperactivity disorder; ABC, Aberrant Behavior Checklist; IQ, intelligence quotient.

Cronbach's alpha was acceptable for the parent ABC hyperactivity subscale for the whole sample and for the ID subgroups (Table 3). None of the items were problematic according to the item‐total correlations and alpha if item deleted for the whole sample and those with mild ID. The item‐total score correlation for *constantly runs or jumps* was low (0.27) amongst those with severe ID, but removing this item showed little improvement in alpha (from 0.89 to 0.90).

#### Teacher measures

For the teacher Conners' hyperactivity scale, high Cronbach's alpha scores were obtained, indicating that the measure was acceptable for the whole sample and when split by ID subgroups (Table 3). None of the items were problematic according to the item‐total correlations or alpha if item deleted for the whole sample or for those with mild ID. Amongst those with severe ID, alpha increased from 0.92 to 0.93 when *runs or climbs excessively* was removed, therefore resulting in minimal improvement. Cronbach's alpha was also acceptable for the teacher Conners' ADHD index for the whole sample and by ID subgroups. None of the items were problematic according to the item‐total correlations or alpha if item deleted for the whole sample or for those with severe ID. For those with a severe ID, alpha increased from 0.93 to 0.94 when the *interrupts or intrudes* item was removed.

For the teacher ABC hyperactivity subscale, Cronbach's alpha was acceptable for the whole sample and when broken down by ID subgroups. None of the items were problematic according to the item‐total correlations or alpha if item deleted for the whole sample or for the different ID subgroups.

### Item response theory

#### Parent measures

The severity, discrimination ability and item information function (IIF) for the parent‐reported Conners' hyperactivity scale are displayed graphically in Fig. S1. The item difficulty (severity) and discrimination parameters are in Table S1. *Always ‘on the go’* was the least severe item, and *restless, ‘squirmy’* was the most severe item, meaning that parents would endorse being *always ‘on the go’* even for children with hyperactivity levels towards the lower end of the continuum (trait), but higher levels of hyperactivity were required for parents to endorse *restless, ‘squirmy’*. The slopes of the item characteristic curves (ICC) were similar in terms of their steepness, indicating that for all items, small differences in levels of hyperactivity corresponded to large differences in the likelihood of item endorsement. The most precise item was *hard to control*, with the rest of the items being broadly similar in terms of their precision and peaking close to the average trait level (*θ*). Being *always ‘on the go’* provided the most information for children with lower levels of hyperactivity.

The difficulty and discrimination parameters and ICC and IIF graphs for parent‐reported Conners' ADHD index are presented in Table S2 and Fig. S2. The item *short attention span* was the least severe item. Item discrimination ability was similar for 9 of the 12 items. For the other three items (*attends if interested*; *messy, disorganised*; and *fidgets, squirms*), the slope of the ICC was less steep, indicating that these items did not discriminate levels of ADHD symptoms as well as other items. The ICC clustered together around the average score. Most items provided information around the average level of the trait. The most informative item was *inattentive, easily distracted*, followed by *distractibility* and *short attention span*. *Attends if interested*, *fidgets, squirms* and *messy, disorganised* did not provide much information across the continuum of ADHD scores.

The difficulty and discrimination parameters and ICC and IIF graphs for the parent‐reported ABC hyperactivity subscale are in Table S3 and Fig. S3. *Easily distractible* was the least severe item, and *no attention when spoken to* was the most severe item. Most ICC clustered around the average score, and items were similar in terms of their discriminative ability. ICC slopes were less steep for three items (*impulsive, boisterous*; *constantly runs or jumps*; and *excessively active*), indicating that these items were less discriminating. The item *no attention when spoken to* provided the most information for higher hyperactivity scorers, and *easily distractible* was most precise for lower hyperactivity scorers. The least informative items across the levels of hyperactivity scores were *impulsive*, *constantly runs and jumps* and being *excessively active*. Most of the other items were similar in terms of precision, with a peak close to the average level.

#### Teacher measures

The item difficulty (severity) and discrimination parameters for the teacher‐reported Conners' hyperactivity scale are in Table S4. The ICC and IIF graphs are in Fig. S4. *Excitable, impulsive* was the least severe item, and *difficulty playing quietly* was the most severe item, meaning that teachers would endorse being *excitable, impulsive* for children with lower levels of hyperactivity, but greater levels of the trait were needed for endorsement of the item *difficulty playing quietly*. The slopes of the ICC‐indicated items were similar in terms of discriminative ability and clustered around the average score. Most items were broadly similar in terms of their precision and peaked around the average trait level. Having *difficulty playing quietly* was most informative for those with higher levels of hyperactivity. The least informative item was *leaves seat*.

The difficulty and discrimination parameters and ICC and IIF graphs for teacher‐reported Conners' ADHD index are reported in Table S5 and Fig. S5. The least difficult item was *short attention span*, and the most severe item was *attends if interested*. Most ICCs were clustered around the average score. The slopes of the ICC were similar in terms of their steepness; slopes were less steep for some items, meaning that these were less discriminating (e.g., *attends if interested*; *interrupts or intrudes*; *and fails to finish tasks*). Many items contributed information around the average to above‐average scores. Being *restless* was most precise for those with higher levels of ADHD, and *short attention span* was most informative for lower scorers. *Attends if interested* provided little information across the continuum of the trait.

The difficulty and discrimination parameters and ICC and IIF graphs for the teacher‐reported ABC hyperactivity subscale are in Table S6 and Fig. S6. In terms of discriminative ability, most items were broadly similar in their slope and clustered just above the average scorer. The item *no attention when spoken to* was the least discriminative item and also the most severe item. Precision for most items was high for the average to above‐average scores. Two items (*disobedient* and *disrupts group activities*) provided a lot of information for scorers towards the higher end of the continuum of hyperactivity. Being *easily distractible*, paying *no attention when spoken to* and paying *no attention to instructions* were the least informative items.

### Measurement invariance

#### Parent measures

For the parent‐reported Conners' hyperactivity scale, measurement invariance was demonstrated for five of the seven items. The item *difficulty waiting* was measurement non‐invariant for both IQ and autism severity. Endorsement of the item increased with higher IQ but decreased with greater autism symptoms (Table 4). Endorsement of being *always ‘on the go’* was the only item measurement non‐invariant by child age, with scores decreasing on this item with increasing child age. For the Conners' ADHD index, measurement invariance for IQ scores was demonstrated for all 12 items; only one item was measurement non‐invariant by autism symptoms and one by child age. Parental endorsement of the item *attends if interested* was higher with increasing autism symptoms. There was measurement non‐invariance by child age for *short attention span*, with decreasing endorsement of this item as children get older. For the parent ABC hyperactivity subscale, measurement invariance by IQ scores was demonstrated on all 16 items. For *no attention when spoken to*, there was measurement non‐invariance by autism symptoms, indicating that endorsement increased with more autism symptoms. Age impacted the endorsement of three items, with less endorsement of *constantly runs or jumps* and *excessively active* and greater endorsement of *disturbs others* with increasing child age.

**Table 4:** Results of the MIMIC models showing significant direct effects of IQ scores, autism symptoms and child age on the parent‐reported and teacher‐reported measures

Measure	IQ scores		Autistic symptoms		Age	
Item	Coef (95% CI)	*P*‐value	Coef (95% CI)	*P*‐value	Coef (95% CI)	*P*‐value
Parent						
Conners' hyperactivity scale						
Difficulty waiting	0.06 (0.02, 0.11)	0.006	−0.06 (−0.11, −0.00)	0.042		
Always ‘on the go’					−0.19 (−0.37, −0.02)	0.029
Conners' ADHD index	No significant effects					
Attends if interested			0.05 (0.00, 0.10)	0.045		
Short attention span					−0.30 (−0.53, −0.06)	0.012
ABC hyperactivity subscale	No significant effects					
No attention when spoken to			0.05 (0.00, 0.10)	0.038		
Disturbs others					0.20 (0.03, 0.37)	0.024
Constantly runs or jumps					−0.16 (−0.30, −0.01)	0.036
Excessively active					−0.19 (−0.36, −0.02)	0.028
Teacher						
Conners' hyperactivity scale	No significant effects		No significant effects		No significant effects	
Conners' ADHD index	No significant effects		No significant effects		No significant effects	
ABC hyperactivity subscale	No significant effects				No significant effects	
Disturbs others			0.66 (0.00, 0.13)	0.037		

A positive coefficient indicates that greater endorsement of the item is associated with increasing IQ scores, more autism symptoms and increasing child age. A negative coefficient indicates that greater endorsement of the item is associated with a decreasing IQ score, fewer autism symptoms and a decreasing child age.

MIMIC, multiple indicators, multiple causes; IQ, intelligence quotient; CI, confidence interval; ADHD, attention deficit hyperactivity disorder; ABC, Aberrant Behavior Checklist.

#### Teacher measures

The teacher Conners' hyperactivity scale and ADHD index were measurement invariant by IQ severity, autism symptoms and child age (Table 4). For the ABC hyperactivity subscale, the items were measurement invariant by IQ and age, but autism symptoms influenced the endorsement of one item (*disturbs others*), indicating that endorsement of this item was more likely for those with more autism symptoms.

## Discussion

The current study examined the psychometric properties of two widely used screening instruments for ADHD symptoms amongst a sample of 7 to 15‐year‐olds with co‐occurring ID and ADHD. We explored the internal reliability, item performance and measurement invariance of parent and teacher versions of the revised Conners' Rating Scale hyperactivity scale and ADHD index and the ABC hyperactivity subscale.

The parent‐reported and teacher‐reported Conners' hyperactivity scale and ADHD index and the ABC hyperactivity subscale were internally reliable amongst the current sample. This is in line with previous research reporting acceptable internal reliability for the Conners' hyperactivity scale and ADHD index and the ABC hyperactivity subscale amongst 5 to 12‐year‐olds with ID (Miller *et al*. 2004). Findings from the IRT analysis indicate that the items on all measures, as reported by parents and teachers, were generally performing well in this sample. Items tended to cluster around the average score of the trait, and a variety of items tapping into both problems with attention and hyperactivity/impulsivity were the least/most severe or discriminative, indicating that problems with specific clusters of items were not found. In the current study, most items provided information across the continuum of the trait; however, some items were not contributing much information above and beyond other items (e.g., *attends if interested* for teacher‐reported Conners' ADHD index scores) and may be redundant. Furthermore, specific items gave more information for low/high scorers. This should be considered when interpreting the endorsement of items and whether less or more weight should be placed on specific symptoms. Of note, the patterns of which items were the most discriminating and provided the most information differed from parents to teachers. It may be that this is due to situational specificity or rater bias, but obtaining reports from more than one informant, in line with clinical practice, will provide a more comprehensive understanding of an intellectually disabled child's ADHD presentation.

The MIMIC models exploring measurement invariance tested whether, given the same levels of hyperactivity/ADHD, the probability of item endorsement depended on IQ, level of autism symptomatology and child age. Most items on the measures examined demonstrated measurement invariance by IQ, autism symptoms and child age, meaning that scores can be compared between individuals with these differing characteristics. However, there was some measurement non‐invariance for parent‐reported ADHD symptoms by these characteristics, but where this occurred, most effects were small and in the expected direction. For example, increasing child age was associated with less endorsement of hyperactivity, which has been found to reduce over time in children without ID, while symptoms of inattention tend to remain (Willcutt *et al*. 2012; Wootton *et al*. 2022). Interpreting responses to items related to activity levels in adolescence should be done with caution. Furthermore, a few items on the parent report instruments showed measurement non‐invariance for autism symptoms. Clinicians and researchers should be aware of these effects and may wish to interrogate measurement non‐invariant items in more detail during a clinical or research interview. With the changes in diagnostic criteria from DSM‐IV to DSM‐V, which for the first time allowed the co‐occurrence of ADHD and autism, it would be important for future studies to include autism diagnostic assessments to further understand the impact of autistic symptoms on ADHD measurement in children with ID. Only one item was found to be measurement non‐invariant for teacher reports, indicating that overall, the teacher endorsement items are similar across the severity of ID, differing levels of co‐occurring autism symptoms and as children get older. This suggests that teacher reports are measuring similar underlying latent traits, regardless of these characteristics.

### Limitations

All the children in the sample were diagnosed with a gold standard ADHD research diagnosis given by experienced clinicians. Therefore, by default, the sample all had high ADHD scores that cluster around the average level of the latent trait but reflect elevated levels. The findings are therefore not generalisable to ID children and young people who do not display significant problems with hyperactivity/impulsiveness and/or inattention, so it is not clear how well these measures perform when used to rule out a possible ADHD diagnosis. It may be that items on these measures may work in a different way for children with ID when levels of the latent trait (ADHD) are low. Adaptive functioning measures were not collected as part of the trial, so it is unknown how impairments in adaptive functioning may have impacted the measurement of ADHD symptoms. In addition, the relatively small sample size, when split into subgroups (e.g., for the reliability analysis), limits the strength of the conclusions that can be drawn, and replication in other samples is needed. Because all of the sample had ADHD, we were also unable to examine the area under the curve, sensitivity and specificity of the measures.

### Strengths and conclusions

This study used a well‐characterised sample of children and young people with ID and co‐occurring ADHD to examine the psychometrics of widely used ADHD screening instruments. The wide range of intellectual functioning and ages of the children and the variation in the co‐occurrence of autism symptoms enabled us to explore whether these key child characteristics impact the measurement of ADHD symptoms. Having reports from both parents and teachers on two measures, one developed for use within the general population and the other for individuals with developmental disabilities, meant that we could replicate patterns of results with different instruments, strengthening the findings. The findings indicate that the parent and teacher revised Conners' Rating Scale hyperactivity scale and ADHD index and the ABC hyperactivity subscale had acceptable internal reliability, adequate item performance and few items that were measurement non‐invariant by IQ, autism symptoms and child age in the current sample of youth with ID.

## Author contributions

ES obtained funding for the original trial. MP, ZF, VCL and ES were involved in designing and conducting the study or analysis. The manuscript was drafted by MP, and all authors read, made revisions and approved the final version.

## Source of funding

The trial was funded by The Health Foundation, formerly the PPP Foundation, reference number 1978, and sponsored by King's College London. MP is supported by a research grant from the Baily Thomas Charitable Fund (TRUST/VC/AC/SG/5841‐8993). VCL is supported by a Sir Henry Wellcome Postdoctoral Fellowship (Wellcome Trust) (213608/Z/18/Z). ES receives support from the National Institute for Health and Care Research through a Senior Investigator Award (NF‐SI‐0514‐10073).

## Conflict of interest

All authors declare no conflicts of interest.

## Supporting information


**Table S1.** Item difficulty and discrimination parameters for parent‐reported Conners' hyperactivity scale.
**Figure S1.** ICC and IFF curve graphs for parent‐reported Conners' hyperactivity scale.
**Table S2.** Item difficulty and discrimination parameters for parent‐reported Conners' ADHD index.
**Figure S2**. ICC and IFF curve graphs for parent‐reported Conners' ADHD index.
**Table S3.** Item difficulty and discrimination parameters for parent‐reported ABC hyperactivity subscale.
**Figure S3.** ICC and IFF curve graphs for parent‐reported ABC hyperactivity subscale.
**Table S4.** Item difficulty and discrimination parameters for teacher‐reported Conners' hyperactivity scale.
**Figure S4.** ICC and IFF curve graphs for teacher‐reported Conners' hyperactivity scale.
**Table S5.** Item difficulty and discrimination parameters for parent‐reported Conners' ADHD index.
**Figure S5.** ICC and IFF curve graphs for teacher‐reported Conners' ADHD index.
**Table S6.** Item difficulty and discrimination parameters for teacher‐reported ABC hyperactivity subscale.
**Figure S6.** ICC and IFF curve graphs for teacher‐reported ABC hyperactivity subscale.

## Data Availability

The data that support the findings of this study are available from the corresponding author upon reasonable request.
