# Supplementary material for: Psychometric properties of two ADHD rating scales used in children with ADHD and intellectual disability
Source: J Intellect Disabil Res. 2024 Sep 7;68(12):1408–21. doi: 10.1111/jir.13185 (PMC11804977; doi:10.1111/jir.13185)
Supplement: Supplementary file 1 — Table S1. Item difficulty and discrimination parameters for parent‐reported Conners' hyperactivity scale. Figure S1. ICC and IFF curve graphs for parent‐reported Conners' hyperactivity scale. Table S2. Item difficulty and discrimination parameters for parent‐reported Conners' ADHD index. Figure S2. ICC and IFF curve graphs for parent‐reported Conners' ADHD index. Table S3. Item difficulty and discrimination parameters for parent‐reported ABC hyperactivity subscale. Figure S3. ICC and IFF curve graphs for parent‐reported ABC hyperactivity subscale. Table S4. Item difficulty and discrimination parameters for teacher‐reported Conners' hyperactivity scale. Figure S4. ICC and IFF curve graphs for teacher‐reported Conners' hyperactivity scale. Table S5. Item difficulty and discrimination parameters for parent‐reported Conners' ADHD index. Figure S5. ICC and IFF curve graphs for teacher‐reported Conners' ADHD index. Table S6. Item difficulty and discrimination parameters for teacher‐reported ABC hyperactivity subscale. Figure S6. ICC and IFF curve graphs for teacher‐reported ABC hyperactivity subscale. [file JIR-68-1408-s001.docx]

**Title:** Psychometric properties of two ADHD rating scales used in children with ADHD and intellectual disability

Operationalisation of ADHD diagnostic procedure

Hyperkinetic disorder was assessed by parental interview, undertaken by medical doctors in psychiatry and paediatrics, postgraduate research and clinical psychologists using the Child and Adolescent Psychiatric Assessment (CAPA; Angold, et al., 1995). The CAPA elicits behavioural accounts of hyperkinetic symptoms that were judged for their developmental appropriateness. Prior to completing the CAPA, interviewers gained parental views of the child’s developmental level and used this information to help the parent identify behavioural examples that reflected a developmentally appropriate activity for the child. In addition, psychologists recorded the child’s behaviour during individual assessments of cognition and a free play/structured activities task designed to assess activity, attention and impulsivity with a descriptive commentary. Where teacher Conners’ ratings were at odds with parental accounts and researcher observations, a school observation was completed using a recording sheet to describe overactivity, inattention and impulsivity. A vignette using all sources of information described above was independently rated for ICD-10 criteria. An initial calibration exercise was carried out amongst the senior investigators to establish standards of behavioural examples meeting individual criteria. All subsequent vignettes were rated by at least one senior investigator, with a second opinion in borderline cases. Mood and anxiety disorders were screened to ensure they did not account for the current symptom presentation. Autistic symptoms were quantified using the Social Communication Questionnaire (Rutter et al., 2003) but the exclusion was not applied.

##### Table S1: Item difficulty and discrimination parameters for parent-reported Conners’ hyperactivity scale

|  | **Difficulty parameters** | | **Discrimination parameters** | |
| --- | --- | --- | --- | --- |
|  | **Coefficient** | **Standard error** | **Coefficient** | **Standard error** |
| Always “on the go” | ***-0.15*** | 0.15 | 1.92 | 0.57 |
| Hard to control | 0.46 | 0.15 | **2.33** | 0.71 |
| Runs or climbs excessively | 0.50 | 0.17 | 1.86 | 0.52 |
| Restless, “squirmy” | **0.86** | 0.22 | 1.64 | 0.48 |
| Difficulty waiting | 0.53 | 0.19 | 1.64 | 0.47 |
| Difficulty playing quietly | 0.32 | 0.18 | ***1.56*** | 0.44 |
| *Note.* Bold characters indicate the largest coefficient. Bold italic characters indicate the smallest coefficient.  Item abbreviations for the parent-reported Conners’ hyperactivity scale: Always “on the go” = Is always “on the go” or acts as if driven by a motor; Hard to control = Hard to control in malls or while grocery shopping; Runs or climbs excessively = Runs about or climbs excessively in situations where it is inappropriate; Restless, “squirmy” = Restless in the “squirmy” sense; Difficulty waiting = Has difficulty waiting in lines or awaiting turn in games or group situations; Difficulty playing quietly = Has difficulty playing or engaging in leisure activities quietly | | | | |

|  |  |
| --- | --- |

Figure S1. ICC and IIF curve graphs for parent-reported Conners’ hyperactivity scale.

*Note for Figures S1 to S6.* The steepness in the S-shaped curve in the Item characteristic curve graphs indicates the item discrimination ability (Jabrayilov et al., 2016). The steeper the curve is indicates that little differences in the trait (i.e., hyperactivity/ADHD, x-axis) correspond to large differences in the likelihood of item endorsement (y-axis), making the item more discriminative. The position of the S-shaped curve in the Item characteristic curve graphs across the trait (x axis) indicates the item severity. The further along the trait continuum the curve is (to the left) means that more of the trait (hyperactivity/ADHD) is needed for the item to be endorsed, making the symptom more severe.

The position and how peaked the bell-shaped curves in the Item information curve graph indicate the amount of information provided by the item on the trait continuum. A more peaked, narrow curve means that the item provided precise information for a particular part of the trait, whereas a wider, less peaked curve means that the item provided was less precise, by provided information across the trait continuum.

##### Table S2: Item difficulty and discrimination parameters for parent-reported Conners’ ADHD index

|  | **Difficulty parameters** | | **Discrimination parameters** | |
| --- | --- | --- | --- | --- |
|  | **Coefficient** | **Standard error** | **Coefficient** | **Standard error** |
| Inattentive, easily distracted | -0.18 | 0.13 | **3.60** | 1.05 |
| Short attention span | ***-0.60*** | 0.16 | 2.65 | 0.67 |
| Fidgets, squirms | 0.32 | 0.21 | 1.14 | 0.31 |
| Messy, disorganised | 0.21 | 0.20 | 1.21 | 0.32 |
| Attends if interested | -0.39 | 0.25 | ***0.95*** | 0.28 |
| Distractibility | -0.36 | 0.14 | 2.77 | 0.72 |
| Mental effort task difficulties | -0.16 | 0.17 | 1.47 | 0.37 |
| Distracted when instructed | 0.01 | 0.15 | 2.07 | 0.50 |
| Trouble concentrating | 0.26 | 0.14 | 2.21 | 0.57 |
| Leaves seat | **0.36** | 0.20 | 1.28 | 0.34 |
| Fails to finish tasks | 0.34 | 0.16 | 1.83 | 0.46 |
| Easily frustrated | 0.12 | 0.16 | 1.59 | 0.39 |
| *Note.* Bold characters indicate the largest coefficient. Bold italic characters indicate the smallest coefficient. Item abbreviations for the parent-reported Conners’ ADHD index: Inattentive, easily distracted = Inattentive, easily distracted; Short attention span = Short attention span; Fidgets, squirms = Fidgets with hands or feet or squirms in seat; Messy, disorganised = Messy or disorganised at home or school; Attends if interested = Only attends if it is something he/she is very interested in; Distractibility = Distractibility or attention span a problem; Mental effort task difficulties = Avoids, expresses reluctance about, or has difficulties engaging in tasks that require sustained mental effort (such as schoolwork or homework); Distracted when instructed = Gets distracted when given instructions to do something; Trouble concentrating = Has trouble concentrating in class; Leaves seat = Leaves seat in classroom or in other situations in which remaining seated is expected; Fails to finish tasks = Does not follow through instructions and fails to finish schoolwork, chores or duties in the workplace (not due to oppositional behaviour or failure to understand instructions); Easily frustrated = Easily frustrated in efforts. | | | | |

|  |  |
| --- | --- |
|  |  |

Figure S2. ICC and IIF curve graphs for parent-reported Conners’ ADHD index

##### Table S3: Item difficulty and discrimination parameters for parent-reported ABC hyperactivity subscale

|  | **Difficulty parameters** | | **Discrimination parameters** | |
| --- | --- | --- | --- | --- |
|  | **Coefficient** | **Standard error** | **Coefficient** | **Standard error** |
| Excessively active | 0.21 | 0.16 | 1.78 | 0.41 |
| Boisterous | 0.83 | 0.21 | 1.56 | 0.39 |
| Impulsive | 0.25 | 0.20 | ***1.14*** | 0.30 |
| Restless | 0.15 | 0.14 | 2.11 | 0.49 |
| Disobedient | 0.34 | 0.14 | 2.45 | 0.59 |
| Disturbs others | 0.14 | 0.14 | 2.39 | 0.56 |
| Uncooperative | 0.85 | 0.16 | 2.83 | 0.74 |
| No attention to instructions | 0.71 | 0.17 | 2.00 | 0.48 |
| Disrupts group activities | 0.50 | 0.14 | 2.74 | 0.68 |
| Leaves seat | 0.37 | 0.14 | 2.33 | 0.56 |
| Will not sit still | 0.24 | 0.13 | 2.70 | 0.66 |
| Easily distractible | **-0.33** | 0.14 | 2.60 | 0.63 |
| Constantly runs or jumps | 0.82 | 0.22 | 1.45 | 0.37 |
| No attention when spoken to | **1.38** | 0.22 | **3.01** | 0.93 |
| Excessively active | -0.12 | 0.17 | 1.50 | 0.36 |
| Ignores directions | 0.98 | 0.17 | 2.83 | 0.75 |
| *Note.* Bold characters indicate the largest coefficient. Bold italic characters indicate the smallest coefficient. Item abbreviations for the ABC hyperactivity subscale: Excessively active = Excessively active at home, school, work, or elsewhere; Boisterous = Boisterous (inappropriately noisy and rough); Impulsive = Impulsive (acts without thinking); Restless = Restless, unable to sit still; Disobedient = Disobedient; difficult to control; Disturbs others = Disturbs others; Uncooperative = Uncooperative; No attention to instructions = Does not pay attention to instructions; Disrupts group activities = Disrupts group activities; Leaves seat (e.g., during lesson or training periods, meals, etc.) = Does not stay in seat (e.g., during lesson or training periods, meals, etc.); Will not sit still = Will not sit still for any length of time; Easily distractible = Easily distractible; Constantly runs or jumps = Constantly runs or jumps around the room; No attention when spoken to = Pays no attention when spoken to; Excessively active = Tends to be excessively active; Ignores directions = Deliberately ignores directions | | | | |

|  |  |
| --- | --- |
|  |  |

Figure S3. ICC and IIF curve graphs for parent-reported ABC hyperactivity subscale

##### Table S4: Item difficulty and discrimination parameters for teacher-reported Conners’ hyperactivity scale

|  | **Difficulty parameters** | | **Discrimination parameters** | |
| --- | --- | --- | --- | --- |
|  | **Coefficient** | **Standard error** | **Coefficient** | **Standard error** |
| Restless, “squirmy” | 0.82 | 0.16 | 2.74 | 0.84 |
| Always “on the go” | 1.03 | 0.19 | 2.46 | 0.73 |
| Leaves seat | 1.14 | 0.22 | ***2.00*** | 0.57 |
| Difficulty waiting | 0.97 | 0.19 | 2.22 | 0.64 |
| Runs or climbs excessively | 0.99 | 0.18 | 2.67 | 0.82 |
| Difficulty playing quietly | **1.61** | 0.26 | **2.92** | 1.05 |
| Excitable, impulsive | ***0.56*** | 0.14 | 2.87 | 0.96 |
| *Note.* Bold characters indicate the largest coefficient. Bold italic characters indicate the smallest coefficient.  Item abbreviation for teacher-reported Conners’ hyperactivity scale: Restless, “squirmy” = Restless in the “squirmy” sense; Always “on the go” = Is always “on the go” or acts as if driven by a motor; Leaves seat = Leaves seat in classroom or in other situations in which remaining seated is expected; Difficulty waiting = Has difficulty waiting his/her turn; Runs or climbs excessively = Runs about or climbs excessively in situations where it is inappropriate; Difficulty playing quietly = Has difficulty playing or engaging in leisure activities quietly; Excitable, impulsive = Excitable, impulsive | | | | |

|  |  |
| --- | --- |

Figure S4. ICC and IIF curve graphs for teacher-reported Conners’ hyperactivity scale

##### Table S5: Item difficulty and discrimination parameters for parent-reported Conners’ ADHD index

|  | **Difficulty parameters** | | **Discrimination parameters** | |
| --- | --- | --- | --- | --- |
|  | **Coefficient** | **Standard error** | **Coefficient** | **Standard error** |
| Inattentive, easily distracted | 0.25 | 0.11 | 3.90 | 1.01 |
| Disturbs others | 0.68 | 0.16 | 2.08 | 0.50 |
| Cannot remain still | 0.86 | 0.15 | 3.10 | 0.82 |
| Fidgets, squirms | 0.81 | 0.13 | 3.88 | 1.09 |
| Short attention span | ***0.10*** | 0.11 | 4.80 | 1.41 |
| Attends if interested | **1.34** | 0.32 | ***1.25*** | 0.36 |
| Distractibility | 0.25 | 0.11 | 3.85 | 0.95 |
| Interrupts or intrudes | 1.03 | 0.23 | 1.54 | 0.40 |
| Fails to finish when started | 1.11 | 0.23 | 1.69 | 0.44 |
| Fails to finish tasks | 1.00 | 1.00 | 2.00 | 0.51 |
| Excitable, impulsive | 0.60 | 0.14 | 2.52 | 0.61 |
| Restless | 0.74 | 0.12 | **5.01** | 1.68 |
| *Note.* Bold characters indicate the largest coefficient. Bold italic characters indicate the smallest coefficient.  Item abbreviation for teacher-reported Conners’ ADHD index: Inattentive, easily distracted = Inattentive, easily distracted; Disturbs others = Disturbs other children; Cannot remain still = Cannot remain still; Fidgets, squirms = Fidgets with hands or feet or squirms in seat; Short attention span = Short attention span; Attends if interested = Only attends if it is something he/she is very interested in; Distractibility = Distractibility or attention span a problem; Interrupts or intrudes = Interrupts or intrudes on others (e.g., butts into others’ conversations or games); Fails to finish when started = Fails to finish things he/she starts; Fails to finish tasks = Does not follow through on instructions and fails to finish schoolwork (not due to oppositional behaviour or failure to understand instructions); Excitable, impulsive = Excitable, impulsive; Restless = Restless, always up and on the go | | | | |

|  |  |
| --- | --- |
|  |  |

Figure S5. ICC and IIF curve graphs for teacher-reported Conners’ ADHD index

##### Table S6: Item difficulty and discrimination parameters for teacher-reported ABC hyperactivity subscale

|  | **Difficulty parameters** | | **Discrimination parameters** | |
| --- | --- | --- | --- | --- |
|  | **Coefficient** | **Standard error** | **Coefficient** | **Standard error** |
| Excessively active | 1.04 | 0.14 | 3.35 | 0.92 |
| Boisterous | 0.97 | 0.13 | 3.94 | 1.12 |
| Impulsive | 0.69 | 0.10 | 5.20 | 1.46 |
| Restless | 0.80 | 0.12 | 3.27 | 0.85 |
| Disobedient | 0.98 | 0.10 | 10.02 | 4.68 |
| Disturbs others | 0.65 | 0.10 | 4.04 | 1.02 |
| Uncooperative | 1.11 | 0.12 | 6.26 | 2.29 |
| No attention to instructions | 1.15 | 0.17 | 2.60 | 0.71 |
| Disrupts group activities | 0.84 | 0.10 | 9.24 | 4.14 |
| Leaves seat | 1.10 | 0.13 | 5.02 | 1.63 |
| Will not sit still | 1.04 | 0.13 | 4.26 | 1.27 |
| Easily distractible | ***0.60*** | 0.13 | ***2.43*** | 0.58 |
| Constantly runs or jumps | 1.37 | 0.18 | 3.84 | 1.27 |
| No attention when spoken to | **1.99** | 0.40 | ***2.43*** | 0.93 |
| Excessively active | 1.00 | 0.13 | 3.75 | 1.04 |
| Ignores directions | 1.59 | 0.24 | 3.69 | 1.41 |
| *Note.* Bold characters indicate the largest coefficient. Bold italic characters indicate the smallest coefficient. Item abbreviations for the ABC hyperactivity subscale: Excessively active = Excessively active at home, school, work, or elsewhere; Boisterous = Boisterous (inappropriately noisy and rough); Impulsive = Impulsive (acts without thinking); Restless = Restless, unable to sit still; Disobedient = Disobedient; difficult to control; Disturbs others = Disturbs others; Uncooperative = Uncooperative; No attention to instructions = Does not pay attention to instructions; Disrupts group activities = Disrupts group activities; Leaves seat (e.g., during lesson or training periods, meals, etc.) = Does not stay in seat (e.g., during lesson or training periods, meals, etc.); Will not sit still = Will not sit still for any length of time; Easily distractible = Easily distractible; Constantly runs or jumps = Constantly runs or jumps around the room; No attention when spoken to = Pays no attention when spoken to; Excessively active = Tends to be excessively active; Ignores directions = Deliberately ignores directions | | | | |

|  |  |
| --- | --- |
|  |  |

Figure S6. ICC and IIF curve graphs for teacher-reported ABC hyperactivity subscale

**References**

Jabrayilov, R., Emons, W.H.M. & Sijtsma, K. (2016) Comparison of classical test theory and item response theory in individual change assessment. *Applied Psychological Measurement, 40*, 559–572.
